# Authentic role of ATP signaling in micturition reflex

**DOI:** 10.1038/srep19585

**Published:** 2016-01-22

**Authors:** Kentaro Takezawa, Makoto Kondo, Hiroshi Kiuchi, Norichika Ueda, Tetsuji Soda, Shinichiro Fukuhara, Tetsuya Takao, Yasushi Miyagawa, Akira Tsujimura, Kazumasa Matsumoto-Miyai, Yusuke Ishida, Hiromitsu Negoro, Osamu Ogawa, Norio Nonomura, Shoichi Shimada

**Affiliations:** 1 Department of Neuroscience and Cell Biology, Osaka University Graduate School of Medicine, Osaka 565-0871, Japan; 2 Department of Urology, Osaka University Graduate School of Medicine, Osaka 565-0871, Japan; 3 Department of Urology, Kyoto University Graduate School of Medicine, Kyoto 606-8507, Japan

## Abstract

Adenosine triphosphate (ATP) is a signaling molecule that regulates cellular processes. Based on previous studies of bladder function over the past decade, bladder ATP signaling was thought to have an essential role in the normal micturition reflex. In this study, we performed detailed analyses of bladder function in purinergic receptor-deficient mice using the automated voided stain on paper method and video-urodynamics. Unexpectedly, a lack of P2X_2_ or P2X_3_ receptors did not affect bladder function under normal physiological conditions, indicating that bladder ATP signaling is not essential for normal micturition reflex. In contrast, we found that lipopolysaccharide (LPS) induced markedly high levels of ATP release from the urothelium. In addition, LPS-induced rapid bladder hyperactivity was attenuated in P2X_2_^−/−^ and P2X_3_^−/−^ mice. Contrary to the previous interpretation, our present findings indicate that bladder ATP signaling has a fundamental role in the micturition reflex, especially in bladder dysfunction, under pathological conditions. Therefore, the bladder ATP signaling pathway might be a highly promising therapeutic target for functional bladder disorders. This study newly defines an authentic role for bladder ATP signaling in the micturition reflex.

ATP is a signaling molecule that regulates cellular processes^1^. Research studies of bladder function have implicated ATP released from the urothelium in the activation of bladder afferent nerves^1^,^2^. Cockyane *et al.* reported that ATP was released from the urothelium in response to mechanical stimuli, and mice lacking the purinergic receptors P2X_2_, P2X_3_ or P2X_2/3_ expressed on a subset of bladder afferent nerves, had reduced micturition reflexes and decreased bladder afferent nerve firing in response to bladder distention^3^,^4^,^5^. In addition, *in vitro* electrophysiological experiments have shown that purinergic receptor antagonists reduce the distension-induced firing of bladder afferent nerves^4^,^6^. Based on these previous studies, it was generally believed that bladder ATP signaling is essential for the micturition reflex: that is, stretch-induced urothelial ATP release evokes the firing of suburothelial bladder afferents via P2X_2_, P2X_3_ or P2X_2/3_ receptors and conveys a sense of bladder filling to the central nervous system^7^,^8^,^9^. These studies suggested that bladder ATP signaling has a prominent role in the normal micturition reflex.

## Results

**Figure 1:**
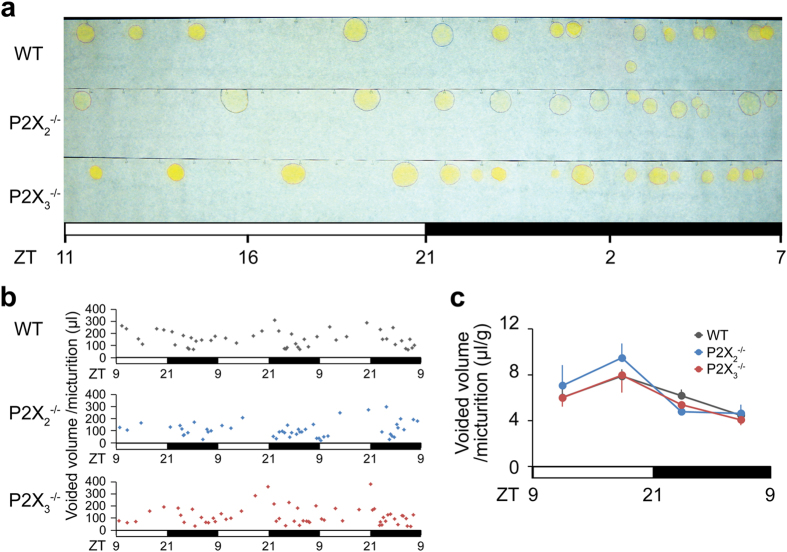
Bladder functional analysis of free-moving WT, P2X_2_^−/−^ and P2X_3_^−/−^ mice in aVSOP. (**a**) Image of urine spots on paper made by WT, P2X_2_^−/−^ and P2X_3_^−/−^ mice. The white and black bars below the graph represent light and dark periods, respectively. ZT, Zeitgeber time. (**b**) Representative charts of voided volume per micturition of WT, P2X_2_^−/−^ and P2X_3_^−/−^ mice for 3 consecutive days. (**c**) Temporal voided volume per micturition every 6 hours. One-way ANOVA and two-way repeated measures ANOVA with Holm correction were used (n = 4, each group). Error bars represent s.e.m.

**Figure 2:**
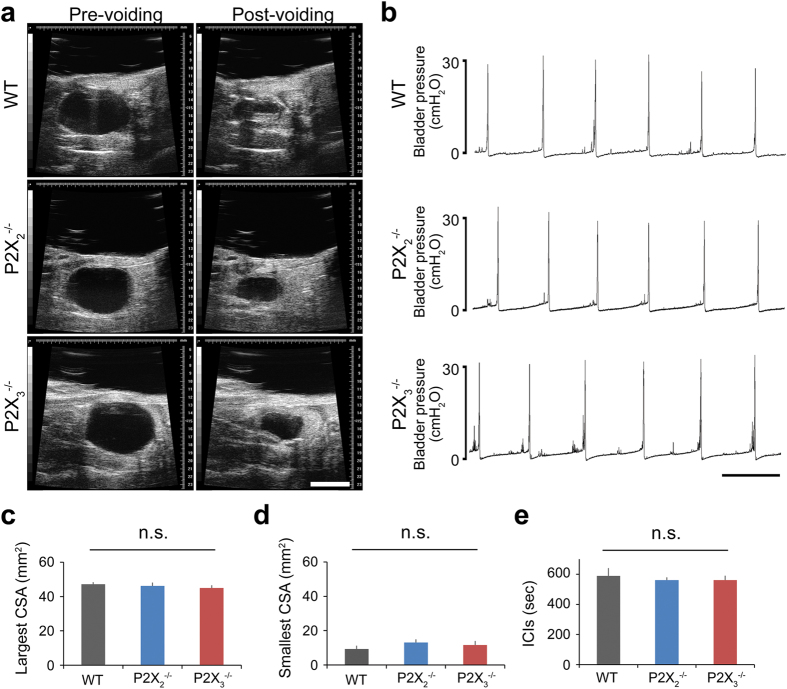
Bladder functional analysis of WT, P2X_2_^−/−^ and P2X_3_^−/−^ mice under normal physiological conditions by video-urodynamics study. (**a**) Transabdominal ultrasonographic findings of voiding in WT, P2X_2_^−/−^ and P2X_3_^−/−^ mice. Scale bar, 5 mm. (**b**) Representative cystometrogram recording charts of WT, P2X_2_^−/−^ and P2X_3_^−/−^ mice. Scale bar, 10 min. (**c–e**) Pre-voiding largest cross-sectional area (CSA) (**c**), post-voiding smallest CSA (**d**) and intercontraction intervals (ICIs) (**e**). The largest and smallest CSA reflect the bladder capacity and the post-voiding residual volume, respectively. One-way ANOVA was used (n = 5, each group). Error bars represent s.e.m., n.s., not significant.

**Figure 3:**
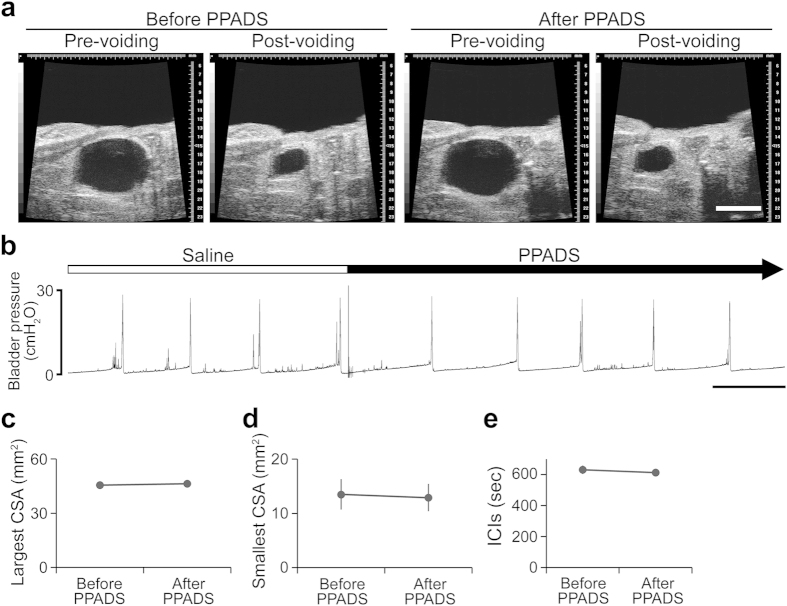
Effects of PPADS on bladder function in WT mice. (**a**) Ultrasonographic findings of voiding before and after PPADS treatment. Scale bar, 5 mm. (**b**) Representative cystometrogram recording chart before and after PPADS administration. Scale bar, 10 min. (**c–e**) PPADS-induced changes of pre-voiding largest CSA (**c**), post-voiding smallest CSA (**d**) and ICIs **(e)**. Paired *t*-test was used (n = 5, each group). Error bars represent s.e.m., n.s., not significant.

To clarify the exact role of bladder ATP signaling in the micturition reflex, we performed *in vivo* functional analysis using P2X_2_^−/−^ and P2X_3_^−/−^ mice (Supplementary Fig. S1). First, bladder function under normal physiological conditions in wild-type (WT), P2X_2_^−/−^ and P2X_3_^−/−^ mice was examined by the automated voided stain on paper (aVSOP) method^10^, which assesses the time of micturition and the voided volume per micturition of free-moving mice for several successive days. There were no significant differences in voided volume per micturition, voiding frequency and total urine volume among WT, P2X_2_^−/−^ and P2X_3_^−/−^ mice (Fig. 1a–c and Supplementary Fig. S2). In addition, to evaluate the mouse bladder function in more detail, we performed a video-urodynamics study, a novel method for the assessment of mouse bladder function, combining cystometry and bladder ultrasonography^11^ (Supplementary Fig. S3). The bladder capacity, post-voiding residual volume, and intercontraction intervals (ICIs) of P2X_2_^−/−^ and P2X_3_^−/−^ mice were similar to those of WT mice (Fig. 2a–e and Supplementary Video S1a–c). Surprisingly, although both P2X_2_^−/−^ and P2X_3_^−/−^ mice were believed to have a considerably larger bladder capacity than WT mice because of their reduced micturition reflex^3^,^4^,^5^, a lack of P2X_2_ or P2X_3_ receptors did not affect bladder function under normal physiological conditions. In addition, intravesical administration of a non-selective purinergic receptor antagonist, PPADS (1 mM), did not affect bladder function in WT mice under normal conditions (Fig. 3a–e and Supplementary Video S2a,b). Our data revealed that bladder ATP signaling is not essential for the normal micturition reflex.

**Figure 4:**
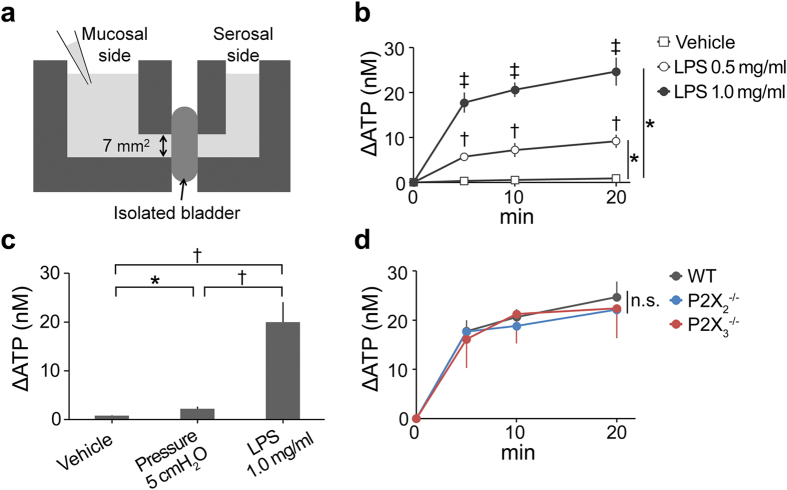
ATP release assay in isolated bladder. (**a**) Schematic of customized Ussing chamber for ATP release assay. (**b**) Time course of ATP concentration (ΔATP) in chamber solution after LPS treatment. *P < 0.001 (time-by-group interaction by two-way repeated measures ANOVA with Holm correction), ^†^*P* < 0.01 versus vehicle and ^‡^*P* < 0.01 versus LPS 0.5 mg/ml (Tukey’s test following one-way ANOVA with Holm correction) (n = 4, each group). (**c**) ΔATP after hydrostatic pressure administration (5 cmH_2_O for 20 min) and LPS treatment (1.0 mg/ml for 20 min). *P < 0.05, ^†^*P* < 0.01 (Student *t*-test with Holm correction following one-way ANOVA) (n = 5, each group). (**d**) Time course of ΔATP after LPS treatment (1.0 mg/ml) in WT, P2X_2_^−/−^ and P2X_3_^−/−^ mice. One-way ANOVA and two-way repeated measures ANOVA with Holm correction were used (n = 4, each group). Error bars represent s.e.m., n.s., not significant.

**Figure 5:**
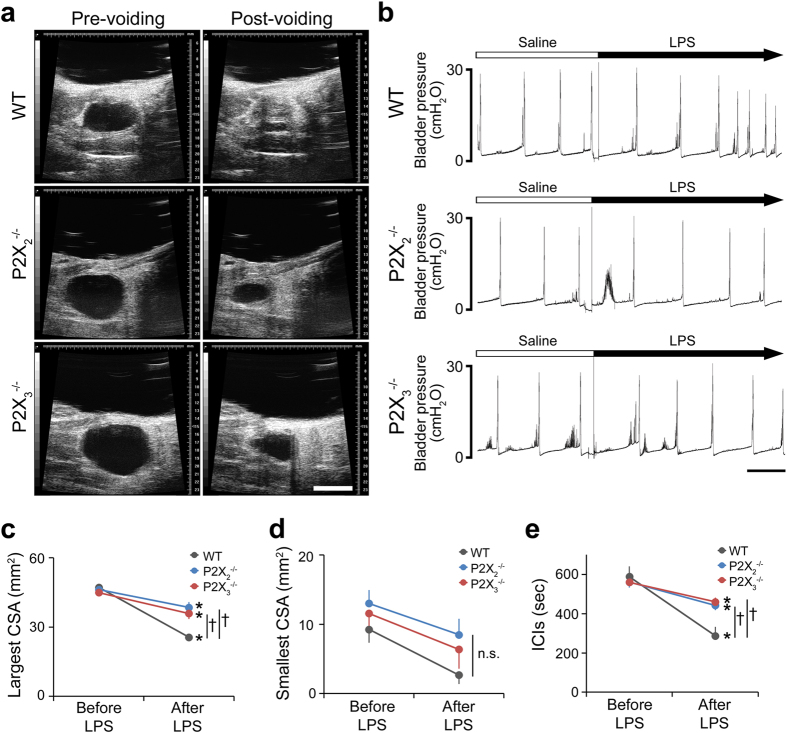
LPS-induced bladder functional changes in WT, P2X_2_^−/−^ and P2X_3_^−/^− mice. (**a**) Ultrasonographic findings of voiding in WT, P2X_2_^−/−^ and P2X_3_^−/−^ mice after LPS instillation. Images were obtained from the same mice after Fig. 2 recording. Scale bar, 5 mm. (**b**) Representative cystometrogram recording charts before and after LPS instillation. Scale bar, 10 min. (**c–e**) LPS-induced changes of pre-voiding largest CSA (**c**), post-voiding smallest CSA (**d**) and ICIs (**e**). *P < 0.05 (paired *t*-test with Holm correction), ^†^*P* < 0.01 (time-by-group interaction by two-way repeated measures ANOVA with Holm correction) (n = 5, each group). Error bars represent s.e.m., n.s., not significant.

**Figure 6:**
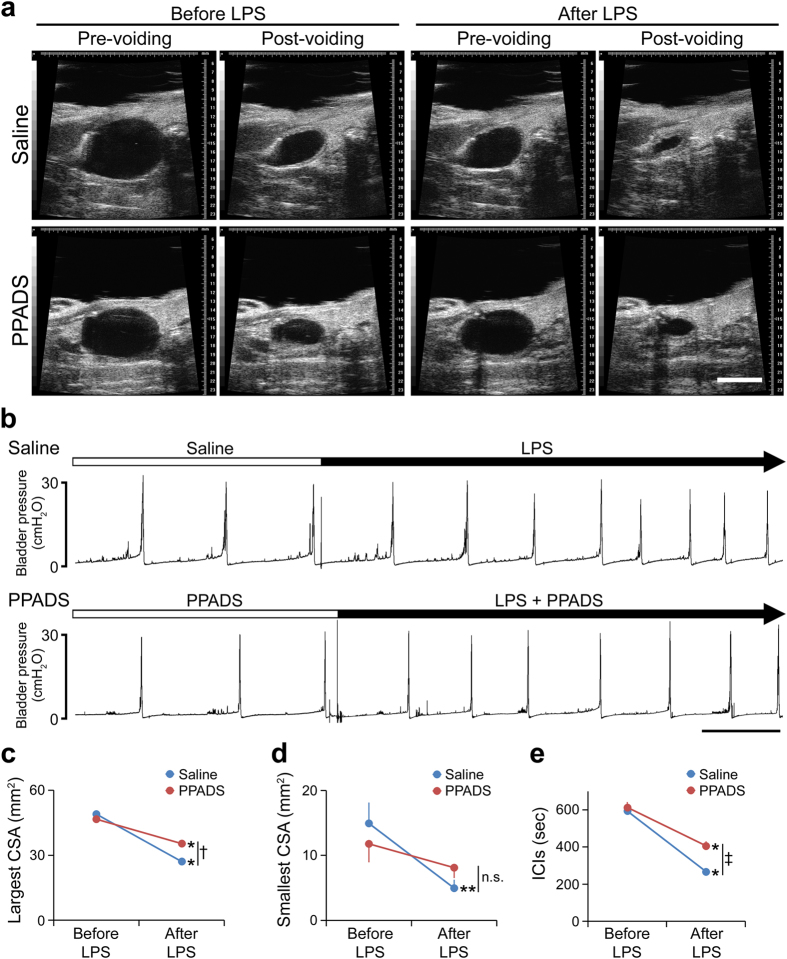
Effects of PPADS on LPS-induced bladder hyperactivity. (**a**) Ultrasonographic findings of voiding in control (Saline group) and PPADS-treated (PPADS group) mice before and after LPS instillation. Scale bar, 5 mm. (**b**) Representative cystometrogram recording charts in control and PPADS-treated mice before and after LPS instillation. Scale bar, 10 min. (**c–e**) LPS-induced changes of pre-voiding largest CSA **(c)**, post-voiding smallest CSA (**d**) and ICIs (**e**) with or without PPADS treatment. *P < 0.01, **P < 0.05 (paired *t*-test with Holm correction), ^†^*P* < 0.01, ^‡^*P* < 0.05 (time-by-group interaction by two-way repeated measures ANOVA) (n = 5, each group). Error bars represent s.e.m., n.s., not significant.

Therefore, what is the authentic role of ATP signaling in the bladder? To answer this we investigated the role of ATP signaling under pathological conditions. Lipopolysaccharide (LPS) is the major component of the outer surface membrane of Gram-negative bacteria, and is used in animal models of inflammation including urinary tract infections^12^. Our previous report revealed that intravesical LPS instillation caused rapid bladder hyperactivity^11^. This rapidity of functional changes implied an unknown mechanism of bladder hyperactivity in response to LPS. Therefore, we examined ATP release from the urothelium following LPS treatment and LPS-induced changes of bladder function in P2X_2_^−/−^ and P2X_3_^−/−^ mice. Results of the ATP release assay in bladders isolated from WT mice^13^ (Fig. 4a) revealed that LPS caused rapid urothelial ATP release that was concentration dependent (Fig. 4b). The level of ATP release induced by LPS was markedly higher than that caused by the distention stimulation (Fig. 4c). The isolated bladders of P2X_2_^−/−^ or P2X_3_^−/−^ mice also showed LPS-induced ATP release comparable to that of WT mice (Fig. 4d). Moreover, bladder functional changes assessed by a video-urodynamics study revealed that intravesical LPS instillation caused a decrease in the bladder capacity and ICIs of WT mice. Importantly, in contrast to WT mice, both P2X_2_^−/−^ and P2X_3_^−/−^ mice showed a significant attenuation of the decreased bladder capacity and ICIs following LPS instillation (Fig. 5a–e and Supplementary Video S3a–c). In addition, intravesical PPADS treatment also attenuated the decrease in bladder capacity and ICIs caused by LPS instillation (Fig. 6a–e and Supplementary Video S4a,b, S5a,b). These results indicated that bladder ATP signaling via P2X_2_ and P2X_3_ receptors has a significant role in rapid bladder hyperactivity induced by LPS.

Together, these results indicated that LPS causes ATP release from the urothelium, and its action on the bladder afferent nerves via P2X_2_ and P2X_3_ receptors is critical for the enhanced micturition reflex. Our findings suggest that bladder ATP signaling has a fundamental role in pathological bladder hyperactivity.

## Discussion

Unexpectedly, our studies revealed that P2X_2_^−/−^ and P2X_3_^−/−^ mice had normal bladder functions under physiological (non-pathological) conditions. This indicated that bladder ATP signaling is not essential for the normal micturition reflex. In the current study we evaluated mouse bladder function using aVSOP and video-urodynamics methods. aVSOP is a novel method for recording free-moving mouse micturition^10^. The diachronic micturition recording of free-moving mice is difficult to measure because of the small volume of urine voided per micturition (sometimes <50 μl). To overcome this problem, we developed a laminated filter paper pretreated to turn the edge of urine stains deep purple and an automated machine called aVSOP. This system enabled an accurate micturition recording of free moving mice fed *ad libitum* for several consecutive days. In addition, video-urodynamics is an innovative method for the detailed analysis of mouse bladder function^11^. In animal models of bladder dysfunction, changes in the maximum bladder capacity and post-voiding residual volume are important for the evaluation of bladder functional changes; however, these changes are difficult to evaluate using traditional methods such as cystometry and measuring voided stains on paper. Previously, we evaluated mouse bladder volume using ultrasonography, and subsequently developed a mouse video-urodynamics method by combining cystometry and transabdominal ultrasonography to enable the simultaneous assessment of bladder pressure and bladder volume^11^. Moreover, we also found that when the bladder catheter for cystometry was conventionally placed in the bladder apex (apex method) it inhibited bladder apex movement and bladder distention. In contrast, a catheter placed in the bladder anterior wall (anterior method) did not inhibit bladder apex movement^11^. The anterior method reflects a more spontaneous micturition than the conventional apex method. Thus, the combination of bladder ultrasonography and anterior method cystometry provides a more accurate and detailed analysis of mouse bladder function than conventional methods. Therefore, our convincing findings that bladder ATP signaling does not contribute to the micturition reflex under non-pathological conditions are based on sound scientific techniques. Previously, Cockayne *et al.* reported that P2X_2_^−/−^, P2X_3_^−/−^ and P2X_2_/P2X_3_^Dbl−/−^ mice had a markedly larger bladder capacity than WT mice by cystometry and electrophysiological analyses^3^,^5^, which is not consistent with our findings by aVSOP and video-urodynamics analysis. The differences in experimental design and procedure may explain this discrepancy. First, the effect of aging on bladder function should be noted. In previous studies of P2X_2_^−/−^ and P2X_3_^−/−^ mice, Cockayne *et al.* used 5- to 6-month-old aged mice^3^,^5^, while 8- to 14-week-old mice were used in this study. Age is one of the most important factors for bladder functional disorders such as overactive bladder and detrusor hyperactivity with impaired contractile function in humans^14^. Another mouse study by Daly *et al.* reported that 24-month-old male mice showed increased voiding frequency and enhanced low threshold afferent nerve activity, compared with 3-month-old control mice, and they showed increased P2X_3_ protein expression and overflow of ATP in the urothelium of aged mice^15^. Thus, aging has a significant effect on mouse bladder function, and bladder ATP signaling may be involved in age-related changes of mouse bladder function. Therefore, the difference in age of mice may explain the discrepancy between our results and those of Cockayne *et al.* Second, the genetic background of the knockout mice might account for some differences because mouse bladder function varies among mouse strains^16^,^17^. In previous studies of P2X_2_^−/−^ and P2X_3_^−/−^ mice, knockout mice with a genetic background of 129Ola × C57BL/6 were used^3^,^4^,^5^. In this study we used P2X_2_^−/−^ and P2X_3_^−/−^ mice with a C57BL/6J genetic background. Therefore, the difference in genetic background of knockout mice might explain the observed discrepancies. Third, the effect of a bladder catheter for cystometry on bladder function might affect results. In our video-urodynamics study, a bladder catheter was placed in the bladder anterior wall, which is different from conventional cystometry (apex method) in which bladder distension was inhibited by the bladder catheter^3^. Our previous study revealed that bladder compliance was significantly decreased using conventional apex method cystometry, resulting in higher bladder pressure during the storage period compared with anterior method cystometry^11^. It was reported that the responses of bladder afferent nerves to bladder pressure were reduced in P2X_2_^−/−^ and P2X_3_^−/−^ mice^5^. Thus, higher bladder pressure during the storage period caused by conventional cystometry might affect the bladder functional analyses of these knockout mice.

Our study had some limitations. First, our study might have been underpowered because of the small sample size. Previous cystometry studies of P2X_2_^−/−^ and P2X_3_^−/−^ used 6–11 mice per group and these knockout mice showed considerably larger bladder capacity than WT mice^3^,^5^. The current study performed aVSOP on four mice and video-urodynamics on five mice per group. However, if the differences among WT, P2X_2_^−/−^ and P2X_3_^−/−^ mice are as considerably significant as reported by Cockayne *et al.*, we should detect significant differences even using our small sample size. Indeed, our study showed comparable bladder function between genotypes under physiological conditions. However, it is possible that there are subtle differences among WT, P2X_2_^−/−^ and P2X_3_^−/−^ mice that were not detected with our sample size. Second, we revealed that bladder ATP signaling via P2X_2_ and P2X_3_ receptors on primary afferent nerves caused by ATP from the urothelium is not essential for the normal micturition reflex; however, the role of ATP signaling in other tissues such as the central nervous system was not examined in this study. Previously, Streng *et al.* reported that the systemic blockade of purinergic receptors with α,β-me-ATP decreased the maximum bladder pressure during the first micturition phase and systemic blockade of both purinergic and cholinergic receptors produced overflow incontinence in rats. They speculated that the purinergic system and cholinergic system might be important for territorial marking and emptying of the bladder, respectively^18^. In rodents, voiding has an ethologically important role including territory demarcation, socialization, reproductive competition, and sexual advertisement^19^,^20^,^21^. Therefore, ATP signaling in the central nervous system might be involved in voiding for these purposes rather than emptying of the bladder, while cholinergic signaling may be responsible for emptying the bladder. Further studies are needed to determine the role of ATP signaling in the central nervous system on the micturition reflex.

Of note, recent studies have raised a controversial question concerning the role of bladder ATP signaling in the micturition reflex. For example, electrophysiological studies showed that a purinergic receptor antagonist, PPADS, did not affect the stretch-induced firing of bladder afferent nerves in guinea pigs^22^,^23^ or rats^24^. In addition, TRPV1 and TRPV4-deficient mice did not exhibit a reduced micturition reflex *in vivo*^25^, although decreased urothelial ATP release was reported in these knockout mice^26^,^27^,^28^,^29^. Crucially, these studies have yielded controversial results related to the significance of bladder ATP signaling in the micturition reflex. Therefore, the exact role of bladder ATP signaling in the micturition reflex has become a fundamental question that needs to be answered to elucidate the physiology of the micturition reflex and the pathophysiology of functional bladder disorders.

Previously, we reported that LPS-induced bladder functional changes were observed early after intravesical LPS instillation^11^. This rapidity of changes in bladder function is reasonable in the light of biological defense reactions to bacterial infection; however, the mechanism involved is unclear. In the present study, we found that LPS induced rapid ATP release from the urothelium of WT mice, which was comparable between P2X_2_^−/−^, P2X_3_^−/−^ and WT mice. Meanwhile, the video-urodynamics study revealed that decreased bladder capacity and ICIs following intravesical LPS instillation were attenuated in P2X_2_^−/−^ and P2X_3_^−/−^ mice, compared with WT mice. These results indicated that LPS-induced increase in urothelial ATP release causes rapid bladder hyperactivity via P2X_2_ and P2X_3_ receptors expressed on bladder afferent nerves. Taken together, the ATP-mediated mechanism of LPS-induced rapid bladder hyperactivity might be a defensive mechanism against bacterial infection of the bladder.

Previous studies have shown that intravesical PPADS instillation ameliorated bladder dysfunction caused by muscarinic acetylcholine receptor agonist^30^ and H_2_O_2_^31^ in rats. In addition, systemic administration of P2X_3_ antagonists, A-317491 or PPADS, attenuated cyclophosphamide-induced bladder hyperactivity in rats^32^. Several clinical reports have shown an increase in ATP release from the urothelium of patients with functional bladder disorders, such as interstitial cystitis and overactive bladder^33^,^34^,^35^,^36^. Thus, regulation of the bladder ATP signaling pathway is considered a potential therapeutic target for functional bladder disorders. In this study, we revealed that bladder ATP signaling has a fundamental role in the micturition reflex under pathological conditions, but not physiological conditions. We found that LPS causes considerably higher levels of urothelial ATP release, compared with distention stimulation. Therefore, our findings suggest that distention stimulation certainly induces ATP release from the urothelium under physiological conditions, but this stimulation is insufficient to enhance the micturition reflex. On the other hand, the large amount of urothelial ATP release induced by LPS is sufficient to cause bladder hyperactivity. Considering these previous reports and our new findings that bladder ATP signaling is not essential for the normal micturition reflex, the regulation of bladder ATP signaling by the blockade of purinergic receptors and inhibition of urothelial ATP release, might be a highly promising therapeutic strategy for functional bladder disorders.

## Methods

### Animals

Experiments were conducted on 8- to 14-week-old male C57BL/6J mice. P2X_2_^−/−^ mice (B6.129-*P2rx2*^*tmlCkn*^/J)^5^ and P2X_3_^−/−^ mice (B6.129P3-*P2rx3*^*tmlCkn*^/J)^3^ were obtained from the Jackson Laboratory (Bar Harbor, ME, USA). These mice were backcrossed onto the C57BL/6J background more than 10 times. Before experiments, mice were housed under a 12:12-h light-dark cycle with controlled humidity and temperature and received food and water *ad libitum*. All procedures were conducted in accordance with the Institution Animal Care and Use Committee of Osaka University. In addition, all experimental protocols were approved by the Institution Animal Care and Use Committee of Osaka University. All efforts were made to minimize animal suffering and reduce the number of animals used.

### Micturition analysis of free-moving mice

Micturition analysis of free-moving mice was performed according to a previously published method^10^. In brief, rolled laminated filter paper was wound up at a speed of 10 cm per hour under a water-repellent wire lattice. Mice were kept in a cage (110 × 160 × 75 mm (height × depth × width)). After an acclimation period of 3 days in the cage, data were continuously collected for each mouse over 3 days. Urine stains were counted, traced, scanned, and quantified by Image J 1.42 software to determine the micturition volume. The formula to produce a standard curve was calculated by correlation with normal saline. The evaluated parameters were voided volume per micturition (μl), voiding frequency per 6 h (times/6 h) and total urine volume per body weight × 6 h (μl/g × 6 h).

### Video-urodynamics study of mice

Analysis of mice by video-urodynamics was performed according to a previously published method^11^. In brief, mice were anesthetized with urethane (1.2 g/kg body weight, intraperitoneally), and the urinary bladder was exposed by way of a lower abdominal midline incision. A polyethylene catheter was inserted through the bladder anterior wall and secured. The catheter was connected with a T stopcock to an infusion pump and a pressure transducer for the measurement of intravesical bladder pressure. Mice were placed in the supine position, an ultrasonography probe was placed on the lower abdomen, and the maximum sagittal cross-section of the bladder was visualized (Supplementary Fig. S3). Bladder ultrasonography was performed with a Vevo 770 Imaging System equipped with a 25-MHz transducer (Visual Sonics, Toronto, ON, Canada). Recordings were started 6 h after surgery. The bladder was infused with saline at 1.5 ml/h. After observation of a stable baseline, mouse bladder function under normal physiological conditions was assessed. Then, 1.0 mg/ml LPS (dissolved in saline) was instilled at 1.5 ml/h. Forty minutes after LPS instillation, LPS-induced functional changes were evaluated. In antagonist studies, a non-selective purinergic receptor antagonist, PPADS (Sigma, St Louis, MO, USA) (1 mM, dissolved in saline) was instilled at 1.5 ml/h for 1 hour followed by LPS (1 mg/ml) and PPADS (1 mM) solution instillation (1.5 ml/h). PPADS-induced functional changes were evaluated at 1 hour after PPADS instillation, and LPS induced functional changes were evaluated at 40 minutes after LPS instillation. The parameters measured by video-urodynamics study were largest cross-sectional area of the bladder (CSA), smallest CSA, and ICIs. Largest CSA and smallest CSA reflected the bladder capacity and post-voiding residual volume, respectively^11^. LPS from *Escherichia coli* 0111:B4 purified by phenol extraction (Sigma) was used. Recordings of intravesical bladder pressures were made using PowerLab and Chart 5 (AD Instruments, Castle Hill, Australia).

### ATP release assay

The urothelial ATP release assay was performed as described previously^13^. In brief, isolated bladders were opened vertically, and mounted to act as a 7-mm^2^ diaphragm between the two halves of a customized small Ussing chamber (Fig. 4a). The mucosal side of the chamber had a volume of 700 μl. Chambers were filled with Krebs solution (117 mM NaCl, 5.9 mM KCl, 1.2 mM MaCl_2_, 24.8 mM NaHCO_3_, 1.2 mM NaH_2_PO_4_, and 11.1 mM glucose) including 2.5 mM CaCl_2_ with 95% O_2_/5% CO_2_ bubbling. In the LPS stimulation assay, LPS from *E. coli* 0111:B4 purified by ion-exchange chromatography (Sigma) was diluted to 0.5 or 1.0 mg/mL in Krebs solution and added to the mucosal side of the chamber. In the distention stimulation assay, hydrostatic pressure at 5 cmH_2_O was applied to the serosal side of the chamber, which reflects the physiological range of bladder pressure threshold for inducing micturition in mice^11^,^13^,^25^. The mucosal side chamber solution was sampled (50 μL) before and after stimulation, and the ATP content was assayed using the luciferin-luciferase method according to the manufacturer’s protocol (Kikkoman, Chiba, Japan). ATP release was determined by subtracting the ATP concentration before stimulation from the concentration after stimulation. The concentration of ATP was determined using standard curves, which were constructed for each experiment using 3 × 10^−7^, 3 × 10^−8^, 3 × 10^−9^, and 3 × 10^−10 ^M ATP.

### cDNA preparation and RT-PCR

Mice were sacrificed by an overdose of pentobarbital. The L6 dorsal root ganglia were removed^37^ and total RNA was extracted according to the manufacturer’s instruction using the RNeasy Mini kit (Qiagen, Valencia, CA, USA). Reverse-transcription was performed using the Prime Script RT reagent Kit (Takara, Shiga, Japan). Then, cDNA samples were subjected to amplification with AmpliTaq DNA Polymerase (Applied Biosystems, Foster City, CA, USA) in a programmable thermal cycler with denaturation at 94 °C for 30 sec, annealing at 56 °C for 30 sec, and extension at 72 °C for 30 sec for 35 cycles as previously described^38^,^39^. The PCR primers were as follows: P2X_2_ receptor, 5′-ACG CTG TGT ACC CTA TTA CC-3′ and 5′-TGC ACT CTG ATT CAG ACA AG-3′; P2X_3_ receptor, 5′-CAG ACT TCT TCA CCT ACG AGA C-3′ and 5′-GTC ACA TAA TCC GAC ACA TCC-3′; and actin (control), 5′-CTG TAT TCC CCT CCA TCG TG-3′ and 5′-GGT GTT GAA GGT CTC AAA CAT G-3′.

### Statistical analysis

For aVSOP, video-urodynamics and ATP release analyses, groups were compared by Student *t*-test, paired *t*-test, one-way analysis of variance (ANOVA), two-way repeated measures ANOVA with Holm correction for repeated comparisons, and Tukey’s test when applicable. All data are presented as means ± standard errors of the means (s.e.m.), and values of *P* < 0.05 were considered statistically significant. All analyses were performed using JMP version 9.03 software (SAS Institute, Cary, NC, USA). Detailed statistical information was as follows: for Fig. 1c, time-by-group interaction by two-way repeated measures ANOVA and one-way ANOVA with Holm correction; for Fig. 2c–e, one-way ANOVA; for Fig. 3c–e, paired *t*-test; for Fig. 4b, time-by-group interaction by two-way repeated measures ANOVA with Holm correction and Tukey’s test following one-way ANOVA; for Fig. 4c, Student *t*-test with Holm correction following one-way ANOVA; for Fig. 4d, time-by-group interaction by two-way repeated measures ANOVA and one-way ANOVA with Holm correction; for Fig. 5c–e, time-by-group interaction by two-way repeated measures ANOVA and paired *t*-test with Holm correction; and for Fig. 6c–e, time-by-group interaction by two-way repeated measures ANOVA and paired *t*-test with Holm correction.

## Additional Information

**How to cite this article**: Takezawa, K. *et al.* Authentic role of ATP signaling in micturition reflex. *Sci. Rep.* **6**, 19585; doi: 10.1038/srep19585 (2016).

## Supplementary Material

Supplementary Information

Supplementary Video 1

Supplementary Video 2

Supplementary Video 3

Supplementary Video 4

Supplementary Video 5
