# Supplementary material for: Authentic role of ATP signaling in micturition reflex
Source: Sci Rep. 2016 Jan 22;6:19585. doi: 10.1038/srep19585 (PMC4726294; doi:10.1038/srep19585)
Supplement: Supplementary Information [file srep19585-s1.doc]

**Supplementary Information**

**Title: Authentic role of ATP signaling in micturition reflex**

**Authors:** Kentaro Takezawa,† Makoto Kondo,†* Hiroshi Kiuchi, Norichika Ueda, Tetsuji Soda, Shinichiro Fukuhara, Tetsuya Takao, Yasushi Miyagawa, Akira Tsujimura, Kazumasa Matsumoto-Miyai, Yusuke Ishida, Hiromitsu Negoro, Osamu Ogawa, Norio Nonomura, and Shoichi Shimada

†These authors contributed equally to this work.

*Corresponding author: Makoto Kondo

**A List of Supplementary Information**

**Supplementary Figures S1-3 and Figure Legends S1-3**

**Supplementary Video Legends S1-5**

**Supplementary Figures S1-3 and Figure Legends S1-3**

**Supplementary Figure S1**

**
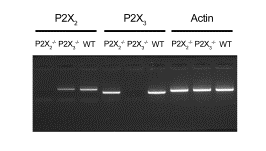
**

**Supplementary Figure S1**

**RT-PCR analysis of P2X_2_ and P2X_3_ receptors from total RNA of L6 dorsal root ganglia of WT, P2X_2_^-/-^ and P2X_3_^-/-^ mice.**

**Supplementary Figure S2**

**
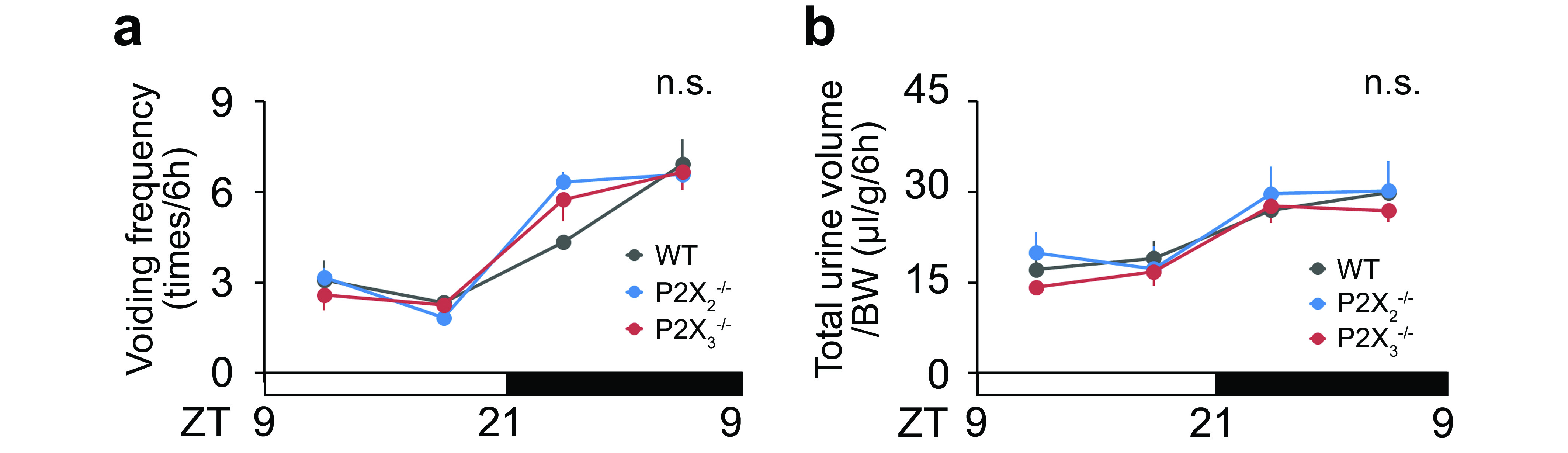
**

**Supplementary Figure S2**

**Voiding frequency and total urine volume in free-moving WT, P2X_2_^-/-^ and P2X_3_^-/-^ mice.**

**(a)** Temporal voiding frequency per 6 h. **(b)** Temporal total urine volume per 6 h. There were no significant differences between WT, P2X_2_^-/-^ and P2X_3_^-/-^ mice. A one-way ANOVA with Holm correction and a two-way repeated measures ANOVA were used (n = 4, each group).

**Supplementary Figure S3**

**
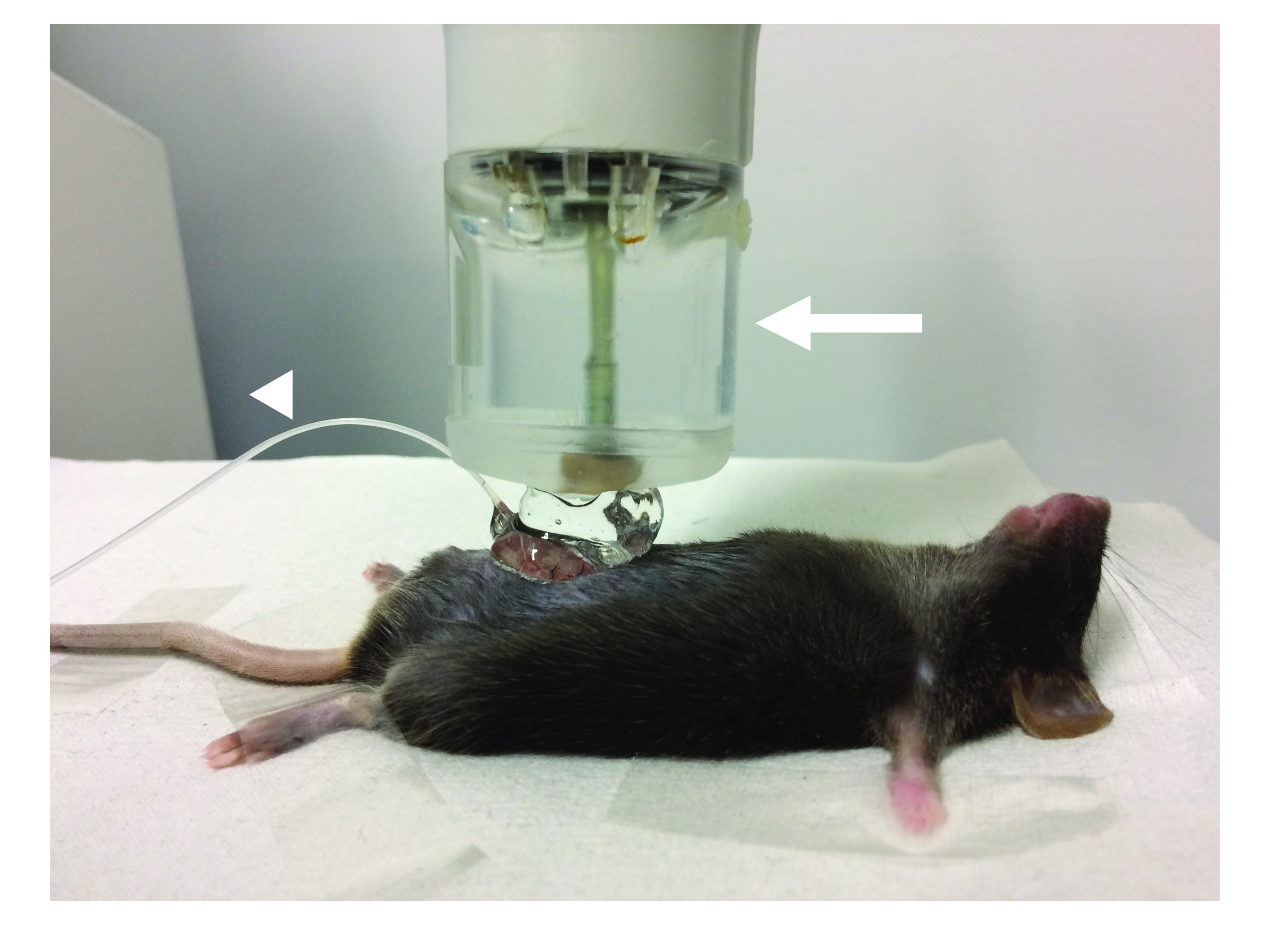
**

**Supplementary Figure S3**

**Image of the mouse video-urodynamics study.**

Arrow indicates the ultrasonography probe. Arrowhead indicates the bladder catheter.

**Supplementary Video Legends**

**Supplementary Video S1**

**Transabdominal ultrasonographic findings of voiding during intravesical saline infusion in WT (a), P2X_2_^-/-^ (b) and P2X_3_^-/-^ (c) mice.**

Capture rate was 10 frames per second. The time lapse covers a period of 10 sec. Frame rate for play is 20 frames per second.

**Supplementary Video S2**

**Transabdominal ultrasonographic findings of voiding before (a) and after (b) intravesical PPADS instillation in WT mice.**

Capture rate was 10 frames per second. The time lapse covers a period of 10 sec. Frame rate for play is 20 frames per second.

**Supplementary Video S3**

**Transabdominal ultrasonographic findings of voiding after intravesical LPS instillation in WT (a), P2X_2_^-/-^ (b) and P2X_3_^-/-^ (c) mice.**

Images were obtained from the same mice as in Supplementary Video 1 recording. Capture rate was 10 frames per second. The time lapse covers a period of 10 sec. Frame rate for play is 20 frames per second.

**Supplementary Video S4**

**Transabdominal ultrasonographic findings of voiding before (a) and after (b) LPS instillation in WT mice.**

Capture rate was 10 frames per second. The time lapse covers a period of 10 sec. Frame rate for play is 20 frames per second.

**Supplementary Video S5**

**Transabdominal ultrasonographic findings of voiding before (a) and after (b) LPS instillation in PPADS-treated WT mice.**

Capture rate was 10 frames per second. The time lapse covers a period of 10 sec. Frame rate for play is 20 frames per second.
